# Novel Insights into Disseminated Candidiasis: Pathogenesis Research and Clinical Experience Converge

**DOI:** 10.1371/journal.ppat.0040038

**Published:** 2008-02-15

**Authors:** Brad Spellberg

**Affiliations:** The Scripps Research Institute, United States of America

Candida spp. have become leading causes of lethal bloodstream infections in countries with advanced medical technology [1]. It is generally and correctly understood that disseminated candidiasis is “an opportunistic infection” that does not occur in healthy people outside of hospitals. It is also incorrectly often said that most patients who develop disseminated candidiasis are “immunocompromised”. While neutropenia (but not lymphocyte dysfunction, including that associated with HIV infection) is indeed a well known risk factor for disseminated candidiasis, patients with neutropenia actually comprise a minority (<20%) of the population that develops disseminated candidiasis [1]. The majority (>80%) of patients who develop disseminated candidiasis are not neutropenic or immunocompromised, and instead have alterations in anatomical barrier function or commensal organism burden due to central venous catheterization, parenteral nutrition, surgical manipulation of the intestines, receipt of broad spectrum antibacterial agents, and/or overgrowth of commensal *Candida* [1]. Furthermore, even when neutropenia is present as a predisposing risk factor, it is typically present because of cancer chemotherapy, which typically also causes disruption of the gastrointestinal mucosal barrier. Hence, the precise role of specific immune dysfunction in predisposing to disseminated candidiasis has been poorly understood to date.

Enter Koh et al. [2]. These investigators have developed a novel murine model of disseminated candidiasis in which gastrointestinal colonization with C. albicans was induced. In contrast, in the standard murine model of disseminated candidiasis, mice are infected via tail vein injection directly into the bloodstream. The standard tail vein model is extremely useful because it accurately recapitulates infection introduced into patients directly through catheters, its clinical course is similar to untreated clinical disseminated candidiasis, and it has been predictive of efficacy of antifungal agents against systemic infection [3–7]. However, an advantage of the novel murine model presented by Koh et al. is that it recapitulates the most common route of infection, translocation of commensal *Candida* across gastrointestinal mucosal surfaces into the bloodstream.

There have been previously established murine models of candidal enteral colonization [8], including a facile model recently published by Clemons at al. [9]. In some of these models immunosuppression of the colonized animals led to dissemination, but the immunosuppression was generally with agents that simultaneously disrupted granulocyte and/or enteric mucosa integrity [8]. Thus, by far the most significant aspect of the current publication is the clever way in which the investigators sequentially disrupted specific host defense elements to determine which protected against disseminated candidiasis from a gastrointestinal source. Consistent with clinical experience and prior murine studies [10,11], the investigators found that depletion of lymphocytes did not predispose to candidal dissemination. Furthermore, even profound depletion of granulocytes (primarily neutrophils) or tissue macrophages was insufficient to enable trans-mucosal dissemination to occur in most animals. These depletion experiments were performed by administering RB6-8C5 antibody (for granulocytes) or liposomal cladronate (for macrophages), and hence spared the enteric mucosa from the damage that normally occurs during myeloablation by chemotherapy. Disruption of enteric mucosal integrity with dextran sulfate was also, by itself, not sufficient to induce disseminated candidiasis. However, when an actual chemotherapy agent (cyclophosphamide) that both ablated neutrophils and also caused gut barrier disruption was administered, lethal disseminated candidiasis developed. Combinations of agents (methotrexate or dextran sulfate + RB6-8C5) that both caused enteric mucosal disruption and depleted granulocytes also led to lethal disseminated candidiasis.

It is surprising that the liver was the primary target organ of infection in the new model because clinical hepatic candidiasis is rare and is typically seen only in the most profoundly immunocompromised patients. Furthermore, in the tail vein model of murine disseminated candidiaisis, the primary target organ of infection is the kidney, hepatic infection occurs at markedly lower levels, and the liver clears infection over time, even at rapidly lethal inocula [4,12]. In another gastrointestinal colonization model in which candidal dissemination was induced by administration of the chemotherapy agent 5-fluorouracil, livers were also more frequently infected than kidneys from days 5 to 15, but by day 15 post-infection kidneys and livers had similar fungal burdens [9]. Whether or not other target organs would become infected at later time points, whether the liver would eventually clear the infection over time, and infection of which organ best correlates with host outcome are questions that merit additional study in this novel model.

Hence, important aspects of clinical disseminated candidiasis have been recapitulated in the novel murine model presented by Koh et al. Furthermore, the model builds upon prior pathogenesis studies [8] and demonstrates that the primary host defense mechanism by which mammals defend ourselves against disseminated candidiasis is intact anatomical surfaces (i.e., gut mucosal barrier and skin). Phagocytes serve as a critical second line of defense against disseminated candidiasis, coming in to play when organisms are able to translocate across damaged anatomical barriers. So, when we say that *Candida* is an opportunistic pathogen, we can now state with confidence that the “opportunity” for the fungus to infect is primarily created by disruption of anatomical barriers and secondarily by abrogation of phagocytic numbers or function.
